# Draft Genome Sequence of the Animal and Human Pathogen *Malassezia pachydermatis* Strain CBS 1879

**DOI:** 10.1128/genomeA.01197-15

**Published:** 2015-10-15

**Authors:** Sergio Triana, Andrés González, Robin A. Ohm, Han A. B. Wösten, Hans de Cock, Silvia Restrepo, Adriana Celis

**Affiliations:** a Department of Biological Sciences, Universidad de los Andes, Bogotá, Colombia; b Grupo de Diseño de Productos y Procesos (GDPP), Chemical Engineering Department, Universidad de los Andes, Bogotá, Colombia; c Microbiology, Department of Biology, Utrecht University, Utrecht, The Netherlands

## Abstract

*Malassezia pachydermatis* is a basidiomycetous yeast that causes infections in humans and animals. Here, we report the genome sequence of *Malassezia pachydermatis* strain CBS 1879, which will facilitate the study of mechanisms underlying pathogenicity of the only non-lipid-dependent *Malasezzia* species.

## GENOME ANNOUNCEMENT

*M. pachydermatis* is the only non-lipid-dependent species of the genus *Malasezzia.* All other 13 species (1) of this genus are obligate lipophilic and require fatty acids for growth. This is due to the lack of a fungal type fatty acid synthase (2). *M. pachydermatis* is able to assimilate fatty acids from the growth medium and can thus be considered a facultative lipophilic species (3). The molecular mechanisms underpinning this behavior are not yet clear.

*M. pachydermatis* is a member of the microbiota of animals. It is an opportunistic pathogen of dogs causing dermatitis and otitis externa. *M. pachydermatis* has also been implicated in human bloodstream infections (4, 5). Three genomes of the obligate lipophilic species *M. globosa*, *M. restricta*, and *M. sympodiales* have been reported (2, 6). Our goal is to understand the facultative lipophilic nature of *M. pachydermatis*.

*M. pachydermatis* genomic DNA was extracted as previously described (7). The DNA was sequenced with the Illumina HiSeq 2000 platform at ServiceXS (Leiden, the Netherlands). Two runs with 120-bp paired-end reads on 250-bp fragments were performed following standard Illumina protocols with a 280-fold genome coverage. Reads were quality controlled with FastQC (8) and trimmed using Flexbar (9). *De novo* assembly was performed using CLC Assembly Cell (CLC bio, Denmark). The resulting contigs were scaffolded using SSPACE_Basic (10), and gaps were filled with GapFiller (11). The ﬁnal assembly consisted of 148 contigs that were linked by pair-end reads into 91 scaffolds, 28 of which were longer than 1 kb. The maximum contig and scaffold length were 1,466,538 and 1,489,072 bp, respectively, and the *N*_50_ was 0.64 Mbp and 1.3 Mbp, respectively. The genome size was 8.15 Mbp with a G+C content of 55.17%.

The genome was annotated using Maker2 (12) and we made use of a set of 109,264 previously reported *Ustilaginomycotina* proteins, 1,413 ESTs from *Malassezia* spp., and CEGMA (13). The homology-based predictor GeneMark and the ab-initio predictors SNAP (14) and Augustus (15) were used to predict genes. In order to train Augustus and SNAP we ran MAKER two consecutive times; the initial annotation output from MAKER was converted into a model for SNAP and a training set for Augustus, which was used in the subsequent run. Functional annotation of the predicted genes was performed by Blast2GO (16), which involved Blast and InterProScan annotation (17, 18).

A total of 4,202 protein-coding genes were predicted with an average size of 1,581 bp. The coding regions corresponded to 81% of the genome. In addition, CEGMA showed that 97.18% of the eukaryotic core genome was present in the genome (13).

Lipid degrading enzymes play an important role in the host invasion process of *M. pachydermatis* (19, 20). A total of 50 lipid degrading enzymes were identified in the genome, including 35 lipases and 15 esterases. Most interestingly, a typical fungal fatty acid synthase was not detected in the genome. Instead a polyketide synthase, homologous to fatty acid synthases (21), was detected that showed 75% identity with its bidirectional homologue of *M. sympodialis*. How *M. pachydermatis* is able to grow in the absence of fatty acids is a subject for future research.

### Nucleotide sequence accession numbers.

This whole-genome shotgun project has been deposited at DDBJ/EMBL/GenBank under the accession number LGAV00000000. The version described in this paper is LGAV01000000.
